# Mechanics of Long-Shank 5 mm Neural Probe Insertion into the Rat Brain: Effects of Geometry and Vibration-Assisted Insertion

**DOI:** 10.3390/mi17060684

**Published:** 2026-05-31

**Authors:** Mahasty Khajehzadeh, Christopher K. Nguyen, Mrigank Maharana, Shriya Peddapuram, Alexandra Joshi-Imre, Juan M. Pascual, Stuart F. Cogan

**Affiliations:** 1 Department of Bioengineering, The University of Texas at Dallas, Richardson, TX 75080, USA; mxk1689@case.edu (M.K.); christopher.nguyen@utah.edu (C.K.N.); mrigank.maharana@utdallas.edu (M.M.); shriya.peddapuram@utdallas.edu (S.P.); 2 Department of Biomedical Engineering, Case Western Reserve University, Cleveland, OH 44106, USA; 3 Department of Electrical and Computer Engineering, University of Utah, Salt Lake City, UT 84112, USA; 4 Office of Research and Innovation, The University of Texas at Dallas, Richardson, TX 75080, USA; alexandra.joshi-imre@utdallas.edu; 5 Division of Child Neurology, Department of Pediatrics, Neurology, Neuroscience and Philosophy, Weill Cornell Medicine, New York, NY 10065, USA

**Keywords:** intracortical microelectrode array (MEA), amorphous silicon carbide (a-SiC), cortical implantation, neural interfaces, dimpling depth, insertion force

## Abstract

Insertion of microelectrode arrays (MEAs) into brain tissue remains a mechanical challenge, especially for long, thin probes designed to access deep structures. This study investigates the mechanical properties of 5 mm long amorphous silicon carbide (a-SiC) probes with different geometries and the effect of vibration-assisted insertion on penetration into rat brain. Methods: Two planar a-SiC probe designs were fabricated with identical lengths and thicknesses but differing width geometries: one with a uniform width (175 µm) and the other with a tapered shape (tapering from 175 to 75 µm). Critical buckling forces (FCs) were estimated by finite element modeling (FEM) and validated experimentally. Insertion mechanics were assessed in a brain mimic of 1.2% agarose gel at varying insertion speeds (20–1000 µm/s) and in vivo by implantation in rat cortex. Insertion metrics included penetration force (F_P_), cortical dimpling depth (D_d_), maximum insertion force (F_max_), and success rate of insertion, all evaluated with and without vibrational assistance. Results: The tapered design exhibited lower penetration force and higher insertion success compared to the uniform-width probe, despite having a lower critical buckling force. An optimal insertion rate of 100 µm/s was identified, balancing insertion time with low F_max_ and high insertion success across designs. Higher F_P_ and D_d_ with a lower success rate were observed for uniform probes compared with tapered probes in rat brain. Vibration-assisted insertion was then investigated with tapered probes. Applying vibration significantly reduced F_P_, whereas D_d_ and F_max_ remained unchanged. Notably, in 40% of actuated insertions in rat, no detectable F_P_ peak was observed, suggesting unimpeded pial penetration. Conclusions: A tapered probe geometry and vibration-assisted insertion can reduce F_max_ and F_P_ while enhancing the insertion success rate for probe penetration in rat brain. These strategies are generally applicable to long-shank MEA insertions in brain and may inform design and insertion strategies.

## 1. Introduction

Multielectrode arrays (MEAs) enable direct interfacing between the brain and external computing systems allowing neural signal acquisition and electrical stimulation [1]. These devices serve as the foundation for brain–machine interfaces (BMIs), with therapeutic applications spanning spinal cord injury, stroke, amyotrophic lateral sclerosis (ALS), sensory restoration, and prosthetic control in amputees [2,3,4,5,6]. In such applications, intracortical MEAs with lengths of approximately 2 mm are often sufficient in length to access the full depth of the cerebral cortex within sulci in porcine models, non-human primates, and humans [3,7,8], or to reach deep-brain targets in rodents. Indeed, preclinical studies, which are often necessary for investigating therapeutic parameters and underlying mechanisms for human applications, are frequently conducted in small animal models such as rats. Whereas clinical DBS targets are located many centimeters below the cortical surface, analogous subcortical structures in rodents are situated within a few millimeters of the cortical surface due to differences in brain size and anatomical scaling. In rats, subcortical regions such as the dorsal striatum, hippocampus, and thalamus are typically located at depths of ~3–5 mm [9,10]. For these targets, the MEA must be capable of reaching these subcortical regions while maintaining mechanical and functional integrity.

Multiple factors, including micromotion, probe stiffness, device architecture, and physical dimensions, influence the long-term stability and functionality of implanted MEAs [11,12,13]. Among these, physical dimensions and cross-sectional area are thought to play an important role in acute insertion trauma and the resulting tissue response [14]. However, reducing probe dimensions also decreases mechanical stiffness [13,15,16], making implantation more difficult due to increased risk of buckling or breakage, especially in longer probes.

A major factor affecting the long-term stability and functionality of implanted MEAs is their physical size and stiffness. Probes with small cross-sectional areas minimize tissue displacement and hemorrhage on implantation and have been shown to reduce foreign body response (FBR) and improve neural recordings with microelectrodes [12,14]. Previous studies have shown that a stiffness mismatch between probe and tissue can exacerbate FBR and contribute to a decline in recording performance. Reducing the cross-sectional area of MEAs decreases the overall mechanical stiffness of intra-parenchymal probe shanks with a consequent minimization of the FBR and improvement in recording stability [11,17,18]. While reducing the cross-sectional area of MEAs can help minimize foreign body response and glial encapsulation [11], it also reduces mechanical stiffness [13,15,16], making implantation more difficult due to increased risk of buckling or breakage, especially in longer probes.

This issue is compounded by the fact that the critical buckling force (F_C_) decreases with increasing probe length, making long and thin shanks highly susceptible to mechanical failure during insertion [15,16]. To address these challenges, several strategies have been proposed to enhance insertion success while minimizing tissue damage. These include optimizing probe geometry—for example, using sharper tip angles [19]; adjusting insertion speed [20]; and, more recently, using vibration-assisted insertion tools [21,22].

In this study, we have extended our previous work on intracortical MEAs [15] and present the design, fabrication, and evaluation of a 5 mm long amorphous silicon carbide (a-SiC) probes aimed at accessing deeper brain regions in rodent models. Amorphous SiC was selected as the structural material of the probe due to its excellent stability and compatibility with thin-film fabrication techniques [23,24,25]. We fabricated and compared two probe designs with identical lengths, thicknesses, and tip angles: one with a uniform shank width and the other with a tapered profile. We used finite element modeling (FEM) to predict F_C_ of both designs and validated model predictions through experimental buckling and tissue insertion measurements. To evaluate insertion performance, we conducted insertion tests in a brain mimic of 1.2% agarose gel [26] to determine the optimal insertion speed based on maximum force and insertion success rate. Finally, we conducted in vivo insertions to determine including penetration force (F_P_), cortical dimpling depth (D_d_), maximum force during implantation (F_max_), and success rate in rat brain, with and without vibration-assisted insertion.

## 2. Materials and Methods

### 2.1. Amorphous SiC Probe Design and Fabrication

**Figure 1:**
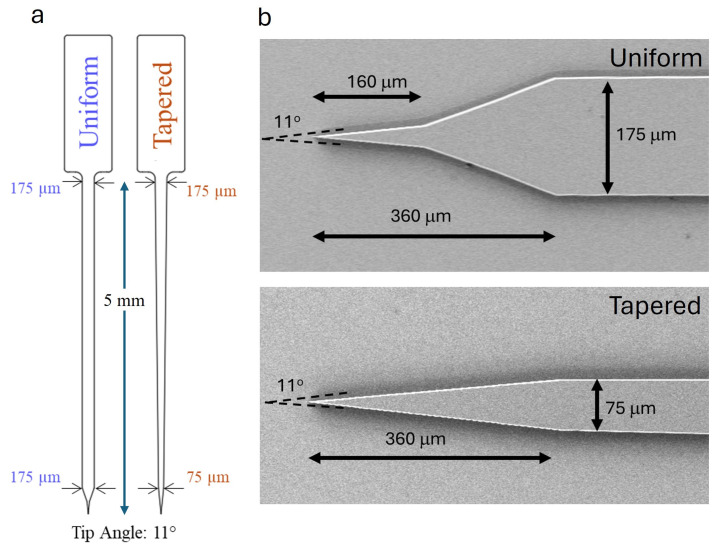
Probe design: (**a**) comparison of uniform and tapered probe geometries; (**b**) scanning electron microscopy (SEM) of tip profiles of tapered and uniform designs defining the 11° tip angle geometry.

Non-functional a-SiC probes with no electrode sites or metal interconnects, but emulating the geometric design of MEAs, were used in this study. As illustrated in Figure 1a we fabricated two probe designs with identical lengths of 5 mm, thicknesses ~12 μm, and tip angle (11°): one with a uniform shank width (175 µm) and the other with a flared profile, tapering from 175 µm at the proximal to 75 µm at the distal tip of the shank. Details of the tip angle geometries for the two designs are compared in the SEM images in Figure 1b. The fabrication process followed previously published methods [15]. Briefly, 1 µm of polyimide (HD Microsystems PI 2610, Parlin, NJ, USA) was spin-coated onto a primed silicon carrier wafer and cured at 350 °C. An 11.5 µm thick layer of amorphous silicon carbide (a-SiC) was deposited by plasma-enhanced chemical vapor deposition (PECVD) at a substrate temperature of 325 °C. Device geometry was defined using photolithography, followed by etching of the a-SiC and underlying polyimide with inductively coupled plasma reactive ion etching (ICP-RIE) using an SF_6_/O_2_ gas mixture. To reduce residual stress, wafers were annealed at 400 °C. Probes were released from the wafer by soaking the wafer in distilled water at 85 °C for several hours.

### 2.2. Critical Buckling Force and FEM

We modeled the effect of probe geometry on F_C_ using finite element modeling (FEM). Whereas the Euler buckling equation provides an analytical estimate of F_C_ for probes with uniform cross-sectional areas (CSAs), this equation is not applicable to tapered probes due to the varying cross-sectional geometry [27]. Therefore, we used a previously validated FEM approach to estimate F_C_ for the tapered design [15]. COMSOL Multiphysics v. 5.2 (COMSOL AB, Stockholm, Sweden) was used to simulate the mechanical behavior, applying fixed–pinned boundary conditions and assuming Young’s modulus as 75 GPa for PECVD a-SiC [28]. To experimentally validate FEM predictions, we measured F_C_ using a previously established method [15]. Probes were mounted onto a 20 g S-beam load cell (FUTEK FSH 3868, Irvine, CA, USA) and connected to a voltage digitizer (FUTEK FSH 03944). Force was recorded as the probe was driven against a rigid surface, a silicon wafer, using the NeuralGlider Inserter (Actuated Medical, Bellefonte, PA, USA) to control the rate of tip advance to the wafer.

### 2.3. Insertion in 1.2% Agarose Phantom

To determine the optimal insertion speed, both probe designs were tested in agarose gel phantoms. Insertion speeds of 20, 100, 500, and 1000 µm/s were evaluated. Similar to methods previously reported [26], we used a 1.2% agarose gel, which has been shown to be a good model for intracortical insertion studies. Briefly, the agarose was prepared by dissolving 0.6 g of agarose powder in 50 mL of deionized water, heating until boiling, and allowing the solution to cool and solidify at room temperature. The same force measurement setup described in Section 2.2 was used. Probes were inserted to a depth of 4 mm from the point of initial contact with the gel surface. The maximum insertion force (F_max_) was defined as the force recorded at 4 mm insertion point.

### 2.4. Insertion in Rat Brain

A total of five adult male rats (Charles River Laboratories, Houston, TX, USA), aged 5 months and weighing 350–450 g, were used. Surgical procedures followed previously established protocols [15,26]. Animals were anesthetized with 2.4–3% isoflurane. The scalp was shaved and disinfected with alternating applications of 70% ethanol wipes and 10% povidone–iodine wipes (3 times). Animals were secured in a Model 942 stereotaxic frame (David Kopf Instruments, Tujunga, CA, USA). A craniotomy was performed, and the dura was carefully removed to expose the cortical surface. Probes were inserted at a single speed of 100 µm/s to a depth of 4 mm from the point of contact with the brain surface. The effect of ultrasonic vibrational actuation of the probe was assessed using a NeuralGlider Inserter with vibration off and on (0.5 W). The vibrational actuation is delivered at a frequency of 20 kHz with a tip displacement of about 5 μm [29].

Three metrics were extracted from the force–displacement curve: F_P_, indicating the force required to puncture the pial surface; D_d_, representing the deformation of the cortical surface prior to penetration of the pia—D_d_ was quantified from the travel distance of the probe holder during insertion, measured from the initial contact of the probe tip with the brain surface to the point of pial puncture and probe penetration into the brain tissue, as identified by the onset of the penetration force (F_P_); and F_max_ as the peak force recorded during full 4 mm insertion. All forces measured in this study are compressive but were reported as absolute positive values.

### 2.5. Statistics

Normality of all datasets was assessed using the Shapiro–Wilk test and visual inspection of Q–Q plots. A one-sample *t*-test was used to compare the FEM-derived F_C_ with the experimentally measured F_C_. One-way ANOVA followed by Tukey’s multiple comparisons test were used to assess the effect of insertion speed in agarose across probe designs. To evaluate the effect of insertion speed within the uniform and tapered designs individually, unpaired *t*-tests were performed for pairwise comparisons between insertion speeds, as the sample size was insufficient to support two-way ANOVA or a mixed-effects model. To evaluate the effects of geometry and actuation on F_P_, D_d_, and F_max_ during brain insertions, one-way ANOVA followed by Dunnett’s multiple comparisons test was performed, with the tapered probe without actuation serving as the control group.

## 3. Results

### 3.1. Critical Buckling Force and FEM

**Figure 2:**
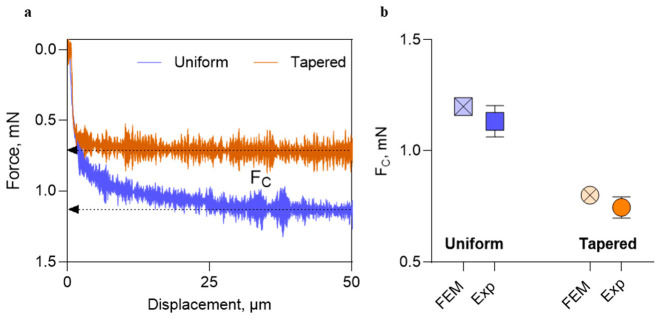
Experimental and predicted buckling behavior of uniform and tapered a-SiC probes. (**a**) Representative force–displacement curves illustrating critical buckling (F_C_) for uniform and tapered geometries. (**b**) Comparison of experimentally measured F_C_ values with FEM predictions (uniform n = 3, tapered n = 4). Arrows indicate the measured F_C_ for each geometry. Data are presented as mean ± standard deviation. No statistical significance was observed between experimental and predicted F_C_ values.

In Figure 2a, representative force–displacement curves for the measurement of F_C_ are shown for both the uniform and tapered probe designs. In each case, the recorded force increased as the probe was advanced against the rigid surface. Upon the onset of buckling, the force reached a plateau, and the maximum value of this plateau was defined as the F_C_. As expected, the probe with a uniform 175 µm width exhibited a higher F_C_ (1.1 ± 0.07 mN, n = 3) than the tapered design (0.7 ± 0.04 mN, n = 4) as shown in Figure 2b. Experimental F_C_ (mean ± SD) values are compared to FEM-predicted values for both designs. A one-sample *t*-test was used to compare the measured values against the FEM-derived reference F_C_. Although the data suggest a slightly lower experimentally measured F_c_ compared with FEM predictions, the differences did not reach significance for either the uniform or tapered probes (uniform: *p* = 0.24, n = 3; tapered: *p* = 0.10, n = 4). These results support the validity of the FEM approach, especially in cases where analytical estimation via Euler’s equation was not feasible due to varying cross-sectional geometry along the shank.

### 3.2. Insertion in Agarose: Effect of Insertion Speed

**Figure 3:**
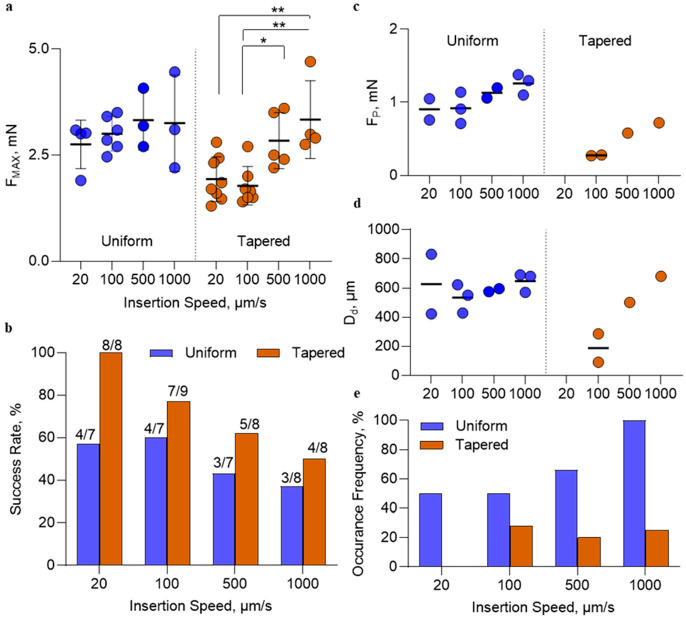
Effect of insertion speed and probe geometry on insertion mechanics in 1.2% agarose. (**a**) Maximum insertion force (F_max_) measured at insertion speeds of 20, 100, 500, and 1000 µm/s for uniform-width (175 µm) and tapered (175–75 µm) probe designs. Horizontal lines indicate mean values and error bars represent standard deviation (SD). Individual points correspond to individual insertion trials. (**b**) Insertion success rate for each probe design as a function of insertion speed. The tapered probe demonstrated higher insertion success across all insertion speeds compared with the uniform probe. (**c**) Penetration force (F_P_) measured in trials where a distinct penetration event was observed. (**d**) Dimpling depth (D_d_) prior to tissue penetration measured for the same trials shown in panel (**c**). (**e**) Occurrence frequency in which F_P_ or D_d_ could be identified across insertion speeds for each probe design. * *p* < 0.05, ** *p* < 0.01.

To enable quantitative comparison of rate on the mechanics of insertion, the maximum insertion force as a function of insertion speed was measured in 1.2% agarose for both probe designs and is plotted in Figure 3a. Horizontal lines represent the mean and error bars indicate standard deviation (SD) for each dataset. For the uniform-width (175 µm) probe, the mean F_max_ was 2.8 ± 0.6 mN (n = 4) at 20 µm/s; 3.0 ± 0.4 mN (n = 6) at 100 µm/s; 3.2 ± 0.7 mN (n = 3) at 500 µm/s; and 3.3 ± 1.1 mN (n = 3) at 1000 µm/s. Using one-way ANOVA, no statistically significant effects of insertion speed on F_max_ were observed in this group (*p* = 0.66). For the tapered probe (175–75 µm), the mean F_max_ values were 1.9 ± 0.5 mN (n = 8), 1.8 ± 0.5 mN (n = 7), 2.8 ± 0.7 mN (n = 5), and 3.3 ± 0.9 mN (n = 4) for insertion speeds of 20, 100, 500, and 1000 µm/s, respectively. Using one-way ANOVA, no significant differences were found between 20 µm/s and either 100 µm/s or 500 µm/s (*p* > 0.09) insertion speeds. However, F_max_ at 1000 µm/s was significantly higher than at 20 µm/s (*p* = 0.007). Significant differences were observed when comparing 100 µm/s with both 500 µm/s (*p* = 0.04) and 1000 µm/s (*p* = 0.003). No significant difference was found between 500 µm/s and 1000 µm/s (*p* = 0.80). To compare the maximum insertion force at each speed between probe geometries (uniform and tapered), an unpaired *t*-test was performed. Significant differences between the uniform and tapered designs was observed at insertion rates of 20 µm/s and 100 µm/s (*p* < 0.03), while no significant differences were observed at 500 µm/s and 1000 µm/s (*p* > 0.36). At slower insertion rates, the viscoelastic agarose matrix has sufficient time to relax, which may contribute to the observed differences between probe geometries. In contrast, at higher insertion rates, the agarose has limited time to relax, resulting in similar maximum insertion forces despite differences in geometric design [30].

The insertion success rate for the uniform probe was 57% (20 µm/s), 60% (100 µm/s), 43% (500 µm/s), and 37% (1000 µm/s), as shown in Figure 3b. A similar decreasing trend with increasing insertion rate was observed for the tapered probes, with success rates of 100%, 77%, 62%, and 50% at 20, 100, 500, and 1000 µm/s, respectively. Overall, the tapered probe demonstrated a higher success rate than the uniform probe across all speeds.

In a subset of trials, an increase in F_P_ and D_d_ in agarose gel could be identified, as shown in Figure 3c,d. In general, both F_P_ and D_d_ increased with insertion speed for both probe geometries, with larger values consistently observed for the uniform probes compared with the tapered design. The frequency of observing these events is summarized in Figure 3e. The number of trials in which F_P_ or D_d_ were observed to increase with insertion speed for the uniform probe was consistently higher than for the tapered design.

Overall, we observe that for the highest success rate and lowest F_max_ for an insertion rate of 20 µm/s, implanting a 4 mm long probe would require more than 3 min, which is impractically long for in vivo surgical procedures. Therefore, considering both insertion reliability and surgical feasibility, an insertion speed of 100 µm/s was selected for subsequent animal experiments.

### 3.3. Insertion in Rat Brain: Effect of Geometry and Actuation

**Figure 4:**
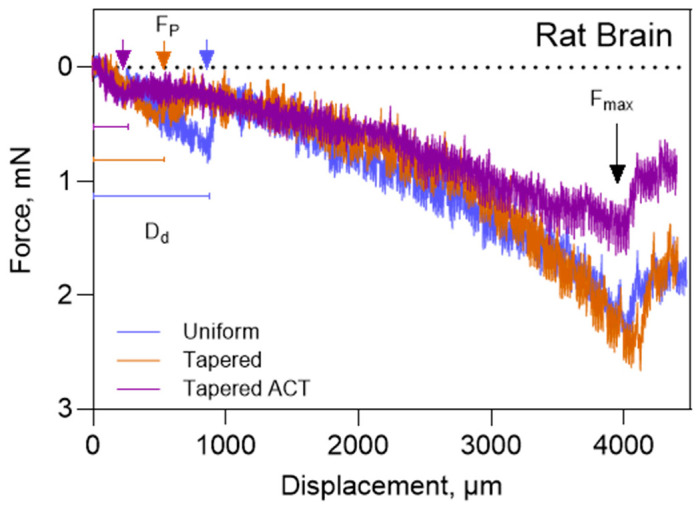
Representative force–displacement curves recorded during insertion of a-SiC probes with varying geometries and actuation condition into rat brain: uniform (175 µm), tapered (175–75 µm), and tapered with vibration-assisted actuation (ACT). The curves illustrate differences in penetration force (F_P_), dimpling depth (D_d_), and maximum force (F_max_) under each condition. Insertion speed is 100 μm/s. F_P_ and D_d_ for each geometry are indicated by colored lines and arrrows matching the figure legend.

Representative force–displacement curves during insertion of the uniform-width probe, the tapered probe without actuation, and the tapered probe with actuation enabled are shown in Figure 4. In all three cases, the insertion speed is 100 μm/s and the initial peak in force corresponds to the puncture of the pial surface (defined as F_P_). Following pial penetration, the insertion force continues to rise as the probe advances into the brain. A maximum insertion force (F_max_) was defined at a 4 mm implantation depth. At this depth, displacement was halted, and the force gradually decreased due to tissue relaxation, consistent with the viscoelastic nature of brain tissue [31]. The length of 4 mm was selected to ensure that the wider portion of the probe at the transition from shank to the external contact pad would not contact the skull and interfere with the measured insertion forces. The most critical stage of implantation is the initial point of entry into the brain tissue; as the probe penetrates deeper into the brain, the shank length remaining outside the brain decreases, which in turn increases F_C_. Consequently, the likelihood of buckling after implantation is reduced.

**Figure 5:**
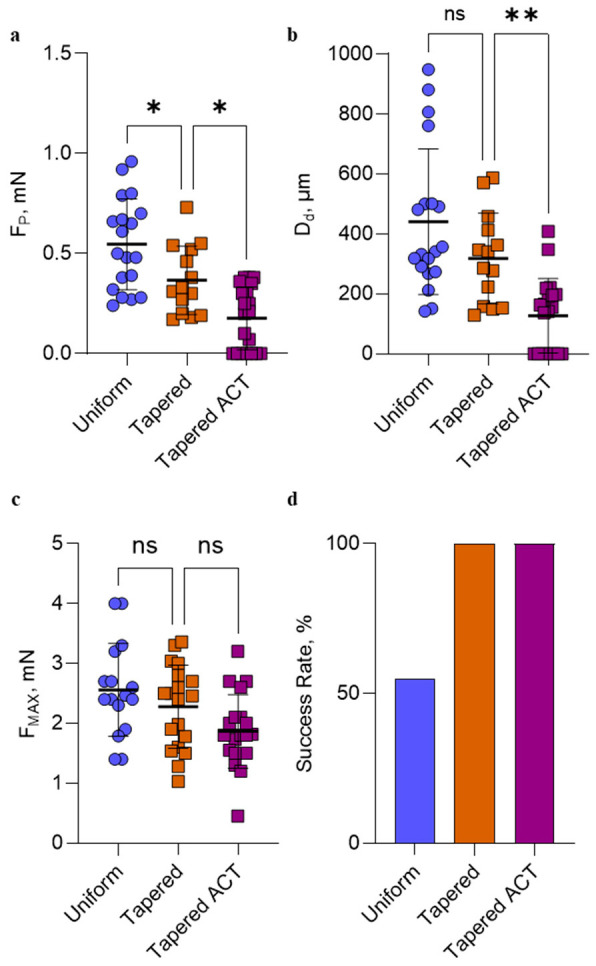
Effects of probe geometry and actuation on insertion mechanics in rat brain tissue. (**a**) Penetration force (F_P_) for uniform-width (n = 19), tapered (n = 14), and tapered probes with actuation (n = 20). (**b**) Dimpling depth (D_d_) prior to cortical penetration for the same three groups. (**c**) Maximum insertion force (F_max_) under each condition. (**d**) Success rate of insertion for each probe geometry and insertion condition. Bold horizontal lines indicate group means and error bars represent standard deviation (SD). Statistical comparisons were conducted using one-way ANOVA followed by Dunnett’s post hoc test when significance was indicated. * *p* < 0.05, ** *p* < 0.01, ns not significant.

In Figure 4, D_d_, F_P_, and F_max_ are indicated on their representative traces. Notably, in this example, insertion of the tapered probe with actuation resulted in near-zero values for both F_P_ and D_d_, indicating minimal resistance from the cortical surface and negligible cortical deformation prior to penetration. The F_P_ across all groups and animals is shown in Figure 5a. The tapered probe without actuation exhibited an F_P_ of 0.37 ± 0.17 mN (n = 14), which was significantly lower than that of the uniform-width (175 µm) probe (0.55 ± 0.23 mN, n = 19; *p* = 0.017). When actuation was applied, the F_P_ for the tapered probe further decreased to 0.18 ± 0.16 mN (n = 20), which was significantly lower than the non-actuated tapered implantation (*p* = 0.010). Figure 5b illustrates the D_d_ for each group. Actuation substantially reduced dimpling during implantation of the tapered probe, from 318 ± 243 µm (no actuation) to 127 ± 124 µm (*p* = 0.007). Although the D_d_ of the tapered probe without actuation was lower than that of the uniform-width probe (440 ± 243 µm), this difference did not reach statistical significance (*p* = 0.1). Notably, in 40% of the insertions performed with the actuated tapered probe, no distinct force peak corresponding to penetration was observed. This suggests that, in these cases, the probe entered the brain tissue without measurable dimpling of the pial, suggesting that vibration-assisted insertion may effectively reduce both the threshold force required to puncture the brain and the degree of surface dimpling, potentially minimizing acute tissue damage at the point of entry.

The F_max_ across all three groups and animals is provided in Figure 5c. The mean F_max_ was 2.5 ± 0.77 mN for the uniform-width probe (n = 16), 2.3 ± 0.70 mN for the tapered probe without actuation (n = 19), and 1.9 ± 0.60 mN for the tapered probe with actuation (n = 20). Although these data suggest a lower F_max_ with actuation, statistically significant difference between groups were not reached. Comparisons were performed to evaluate the effects of probe geometry (uniform vs. tapered) and actuation conditions relative to the tapered design. The insertion success rates are shown in Figure 5d. The tapered probe achieved a 100% success rate both with and without actuation, while the uniform probe exhibited a 55% success rate.

## 4. Discussion

Implantation of microelectrode arrays (MEAs) into neural tissue is inherently invasive and can result in mechanical trauma, leading to a cascade of foreign body responses and eventual formation of glial scarring [12,14,32]. The severity of the acute injury may be a major determinant in long-term MEA performance. In previous work, we demonstrated that a-SiC probes, as mimics for MEAs with intracortical shank lengths of <2 mm and reduced cross-sectional area (CSA), could be implanted with a high insertion success rate in both small and large animals [15]. Building on this foundation, the present study examined the insertion mechanics of longer 5 mm shank a-SiC probes with ~12 µm thickness. Probes with both uniform shank widths (175 µm) and tapered shanks (75 µm at the tip increasing to 175 µm proximally) were investigated. The tapered design offers the advantage of a reduced CSA close to the point of entry, which is expected to decrease F_P_, and a wide proximal shank width that increases bending stiffness to reduce the possibility of buckling as the probe advances into the brain parenchyma. The tapered design has a lower overall F_C_ than the probes with the uniform shank width, although this did not result in a decrease in insertion success rate. Both probe designs share an identical tip angle of 11° (Figure 1). The 5 mm probes were intended to target deep-brain structures in rodents, while the study emphasized understanding the roles of cross-sectional geometry, insertion speed, and probe actuation by ultrasonic vibration and their effects on insertion mechanics and success rate [21]. A major factor affecting the long-term functionality and stability of implanted MEAs is their physical size and stiffness. Probes with small cross-sectional areas that minimize tissue displacement and hemorrhage on implantation and have been shown to reduce foreign body response (FBR) and improve neural recordings with microelectrodes [12,14]. Previous studies have shown that a stiffness mismatch between probe and tissue can exacerbate FBR and contribute to a decline in recording performance. Reducing the cross-sectional area of MEAs decreases the overall mechanical stiffness of intraparenchymal probe shanks with a consequent minimization of the FBR and improvement in recording stability [11,17,18]. Indeed, the meta study by Stiller et al. [13] demonstrated the benefit of reduced probe stiffness in minimizing FBR across a range of microelectrode designs and sizes. Thus, probes fabricated from intrinsically high-elastic modulus materials, such as the a-SiC used in the present study, with small cross-sectional dimensions, as well as larger probes fabricated from low modulus materials may both reduce FBR because of the overall reduction in stiffness.

Implantation of long probes, especially those that are highly flexible to minimize FBR, is challenging due to their vulnerability to buckling as the probe tip contacts the brain surface. Several strategies have been proposed to facilitate insertion while avoiding buckling, among which tip angle and insertion speed are particularly influential [19,33,34]. Although high-speed pneumatic insertion is commonly used to reduce dimpling and tissue displacement in clinical microelectrode devices like Utah-style MEAs [35,36], higher insertion rates can also stiffen the tissue and increase resistance at the point of entry [20,37]. This behavior aligns with prior studies demonstrating that viscoelastic materials, like agarose and brain, stiffen with an increasing strain rate, preventing tissue relaxation during implantation and increasing maximum insertion force [30].

Prior to measurements in the rat model, we evaluated a range of insertion rates from 20 µm/s to 1000 µm/s in agarose gel to determine the effect of speed on the insertion mechanics of the a-SiC probes. The 1.2% agarose gel has similar viscoelastic behavior to rat cerebral cortical tissue [26]. The uniform-width probes did not exhibit significant changes in F_max_ across the 20–1000 μm/s insertion rates investigated (Figure 3a). Although, insertion success rate declined with increasing insertion speed from 78% at 100 µm/s to 56% and 62% at 500 and 1000 µm/s, respectively. Statistical significance could not be tested due to limited sample size. In addition, the uniform-width probe had a higher critical buckling force than the tapered design, despite lower insertion success rate (Figure 3b). For a tapered probe, a marked increase in maximum insertion force (F_max_) was observed at speeds above 100 µm/s in agarose, whereas the uniform-width probe exhibited minimal sensitivity to insertion rate across the same range (Figure 3a). The increase in F_max_ with insertion speed is consistent with prior studies, where higher insertion rates limit tissue relaxation and results in increase resistance during penetration [30].

For both agarose and in vivo rat experiments, tapered probes demonstrated a higher insertion success rate compared to uniform probes, despite having a lower critical buckling force. The aspect ratio of the shank cross-section, defined as the width-to-thickness ratio (w/t), is a critical factor influencing lateral stability during insertion, and probes with the same CSA, but different w/t ratios will exhibit different stability. The uniform-width probe has an aspect ratio of ~15 (175/12), compared to ~6 (75/12) in the tapered design, and is 360 µm proximal to the tip, as shown in Figure 1b. High aspect ratio structures are mechanically more susceptible to lateral deflection, torsion, and off-axis buckling under compressive loading. In contrast, reducing the aspect ratio (i.e., making the cross-section squarer) improves column stability. A more mechanically optimal design would involve increasing thickness while reducing width, thereby lowering the aspect ratio. This modification enhances the second moment of inertia, a geometric property that governs bending resistance, more efficiently than increasing width alone. From a fabrication standpoint, increasing thickness in thin-film MEAs is feasible with adjustments in PECVD or multilayer stacking processes, making this a viable route to improve mechanical robustness. However, increasing the film thickness may elevate the risk of deposition-related defects that are difficult to eliminate [38], and may require alternative annealing strategies to mitigate compressive stress accumulated during the deposition process [39].

Based on our results in agarose, an insertion rate of 100 µm/s was selected for subsequent studies in rat. During implantation in rat brain, the tapered probe design demonstrated significantly lower penetration force (F_P_) compared to the uniform-width probe, despite both probe designs having the same distal tip geometry. This observation is consistent with previous studies showing that penetration force increases with the cross-sectional area (CSA) of the implant [34,40]. In addition, we found that vibration-assisted insertion, enabled through actuation, further reduced F_P_ relative to passive insertion. Actuation at a power level of 0.5 W induces high-frequency (20–25 kHz) axial displacement by 2–5 µm of the probe [29], which alters the insertion mechanics at the probe–tissue interface. Vibrational actuation significantly reduced F_P_ and dimpling depth (D_d_), but it did not produce a statistically significant change in the maximum insertion force (F_max_). This suggests that actuation primarily affects the initial phase of insertion, associated with pial penetration and surface interactions, rather than influencing the deeper mechanical resistance encountered as the probe advances through the parenchyma. Reducing dimpling depth decreases brain tissue deformation before probe penetration. Previous studies suggest that lower dimpling is associated with reduced acute insertion trauma, less hemorrhage, and lower mechanical energy transferred to the tissue hemorrhage [19,41,42]. Therefore, minimizing dimpling depth may help improve the chronic functionality of MEAs intended for long-term implantation. While this study focused on acute mechanical metrics, future work should incorporate histological analysis to evaluate tissue damage, inflammatory response, and glial scar formation. Assessing whether the mechanical advantages of tapered geometries and vibration-assisted insertion also translate to improved chronic biocompatibility is essential for clinical translation. Although the probes used in this study were non-functional, the improved mechanical outcomes, particularly the reduction in dimpling and penetration force, have direct implications for chronic electrophysiology. Reduced insertion trauma is known to correlate with reduced impedance and improved unit yield in chronic animal preparations. Thus, the use of optimized geometries and insertion methods may enhance the recording stability and longevity of functional MEAs in future applications.

## 5. Conclusions

The mechanical insertion of 5 mm long amorphous silicon carbide (a-SiC) probes with different cross-sectional geometries was evaluated in agarose gel and acutely in rat brain. We demonstrated that a tapered probe design significantly reduces penetration force and improves insertion success compared to a uniform-width probe, despite having a lower critical buckling force. Finite element modeling provided accurate predictions of buckling behavior, validating its utility for non-uniform geometries. Insertion rate was found to strongly influence mechanical outcomes, with 100 µm/s identified as the optimal speed for the present study, balancing low insertion force and high success across both designs. Moreover, vibration-assisted insertion further reduced penetration force and cortical dimpling without significantly affecting maximum insertion force. These results highlight the advantages of combining geometric optimization with actuation-assisted techniques to improve the reliability of long-shank MEA implantation. Together, our findings offer design and methodological guidelines that may facilitate safer and more effective deployment of flexible low CSA neural probes for microelectrode targeting of deeper structures in brain recording and stimulation applications. Future work should include chronic implantation and histological analysis to correlate mechanical metrics during insertion with long-term tissue response and the stability of neural recordings.

## Data Availability

The datasets presented in this article are available on request from Stuart F. Cogan and Mahasty Khajehzadeh.

## Abbreviations

The following abbreviations are used in this manuscript:

MEA	Microelectrode array
A-SiC	Amorphous silicon carbide
F_C_	Critical buckling force
F_P_	Penetration force
D_d_	Dimpling depth
F_max_	Maximum force
BMI	Brain–machine interface
FEM	Finite element modeling
